# Acute fibrinous and organizing pneumonia associated with influenza A/H1N1 pneumonia after lung transplantation

**DOI:** 10.1186/1471-2466-13-30

**Published:** 2013-05-19

**Authors:** Claudia Otto, Daniela Huzly, Lars Kemna, Annegret Hüttel, Christoph Benk, Siegbert Rieg, Till Ploenes, Martin Werner, Gian Kayser

**Affiliations:** 1 Institute of Pathology, University Medical Center Freiburg, Breisacher Strasse 115a, Freiburg, D-79106, Germany; 2 Department of Virology, University Medical Center Freiburg, Hermann-Herder Strasse 11, Freiburg, D-79104, Germany; 3 Department of Radiology, University Medical Center Freiburg, Hugstetter Strasse 55, Freiburg, D-79106, Germany; 4 Department of Pneumology, University Medical Center Freiburg, Hugstetter Strasse 55, Freiburg, D-79106, Germany; 5 Department of Cardiovascular Surgery, University Medical Center Freiburg, Hugstetter Strasse 55, Freiburg, D-79106, Germany; 6 Department of Thoracic Surgery, University Medical Center Freiburg, Hugstetter Strasse 55, Freiburg, D-79106, Germany; 7 Department of Internal Medicine, University Medical Center Freiburg, Hugstetter Strasse 55, Freiburg, D-79106, Germany

**Keywords:** AFOP, Influenza A/H1N1, Acute lung failure, Lung transplantation, Viral infection

## Abstract

**Background:**

Immunocompromised patients, particularly after lung transplantation, are at high risk to develop atypical forms of pulmonary infections including influenza A/H1N1. Acute Fibrinous and Organizing Pneumonia (AFOP) is a special histological pattern in acute respiratory failure with high mortality.

**Case presentation:**

We describe a 66-year-old woman with double lung transplantation in August 2009 due to end stage pulmonary fibrosis. After prolonged weaning and subsequent promising course, she developed atypical pneumonia with diffuse pulmonary infiltrates in both lungs in January 2010. Infection with influenza A/H1N1 virus was verified. The patient rapidly suffered from respiratory insufficiency and died eight days after this diagnosis. The post-mortem revealed especially in the lower parts of the lungs the classical histological pattern of pure AFOP. Molecular analyses of lung tissue were positive for influenza A/H1N1.

**Conclusion:**

To our knowledge we present the first case of AFOP triggered by viral infection, here proven to be influenza virus A/H1N1. Thus, also in the setting of viral infection the highly deadly differential diagnosis of AFOP must be considered.

## Background

Immunocompromised patients, especially after lung transplantation are at high risk to develop atypical pneumonias. Among viral infections, influenza A/H1N1 pneumonia can lead to severe respiratory conditions in this patient group often resulting in deadly respiratory insufficiency. Acute fibrinous and organizing pneumonia (AFOP) is a distinct reaction pattern in acute respiratory failure with high mortality rate of upto 90%. Although some causes of AFOP are known so far, its pure form without the presence of accompanying hyaline membranes typical for diffuse alveolar damage (DAD) has not been described to be linked to viral pneumonias.

## Case presentation

### Clinical course

The 66-year old caucasian female patient suffered from end-stage pulmonary fibrosis caused by usual interstitial pneumonia (UIP) first diagnosed in November 2004. According to the guidelines of the International Society for Heart and Lung Transplantation, the patient was submitted to double sided lung transplantation in August 2009. Immediate post-operative course was complicated by prolonged respiratory weaning with the necessity of percutaneous tracheotomy. The patient could not be immunized with influenza vaccination due to poor general condition in September and October 2009, the vaccination period for the seasonally expected influenza viruses. In November 2009, the patient was transferred to rehabilitation treatment. During the end of December 2009 and the beginning of January 2010, she complained about persistent cough. Because of aggravation of her general and especially respiratory condition, she was admitted to a local district hospital and, due to respiratory deterioration, subsequent intubation was needed. High-dose corticosteroid therapy was initiated to overcome acute organ rejection. As respiratory insufficiency further deteriorated, she was finally transferred to the intensive care unit of the medical department at the University Medical Center Freiburg.

Nasotracheal secretion was positive for viral influenza A/H1N1 RNA, proved by RT-PCR. Under the working diagnosis of influenza A/H1N1 pneumonia with bacterial superinfection, therapy with meropenem, clarithromycin and oseltamivir was initiated. Because a mutation causing resistance against Oseltamivir was detected.in further PCR work-up, oseltamivir was subsequently switched to zanamivir. Immunosuppressive therapy was continued with prednisolone and mycophenolate-mofetil, while tacrolimus was interrupted due to high serum levels.

Because of acute renal failure and suspected heparin-induced thrombocytopenia type II, continuous venous-venous hemofiltration was initiated. Despite extracorporeal interventional lung assist implantation, sufficient carbon dioxide elimination could not be reached and the patient died due to rapidly progressive respiratory failure four days after the diagnosis of influenza A/H1N1 infection of the lung (timeline of the clinical course see Table 1).

**Table 1:** Timeline of the clinical course

**Date**	**Clinical course**	**Therapy**
November 2004	Diagnosis of usual interstitial pneumonia (UIP)	
August 2009		Double lung transplantation, complicated postoperative course
November 2009	Rehabilitation treatment	
December 2009 - January 2010	Persistent cough	Admission to general hospital
3.1.2010	Aggravation of general/ respiratory condition	Admission to intensive care unit
	Respiratory insufficiency	Intubation
5.1.2010	Nasotracheal secretion positive for viral influenza A/H1N1 RNA (working diagnosis: H1N1 pneumonia with bacterial superinfection)	Meropenem, clarithromycin and oseltamivir
7.1.2010	Detection of viral influenza A/H1N1 RNA by real-time PCR (bronchoscopy)	
	Detection of mutation causing resistence against oseltamivir	Switch from oseltamivir to zanamivir
	Acute renal failure	Continuous venous-venous hemofiltration
8.1.2010	Respiratory insufficiency	Extracorporeal interventional lung assist implantation
11.1.2010	Rapidly progressive respiratory failure	Death

### Bronchoscopy

After admission to the medical intensive care unit, bronchoscopy was performed to evaluate initial differential diagnoses of acute graft rejection and infectious pneumonia. Bronchial mucosa was edematous and reddened. Mucus was viscous but not extensive in amount. Surgical anastomoses were inconspicuous. No pus or signs of acute bacterial infection could be observed. Bronchioalveolar lavage (BAL) fluid and sputum were sent to microbiological examination.

### Imaging

**Figure 1:**
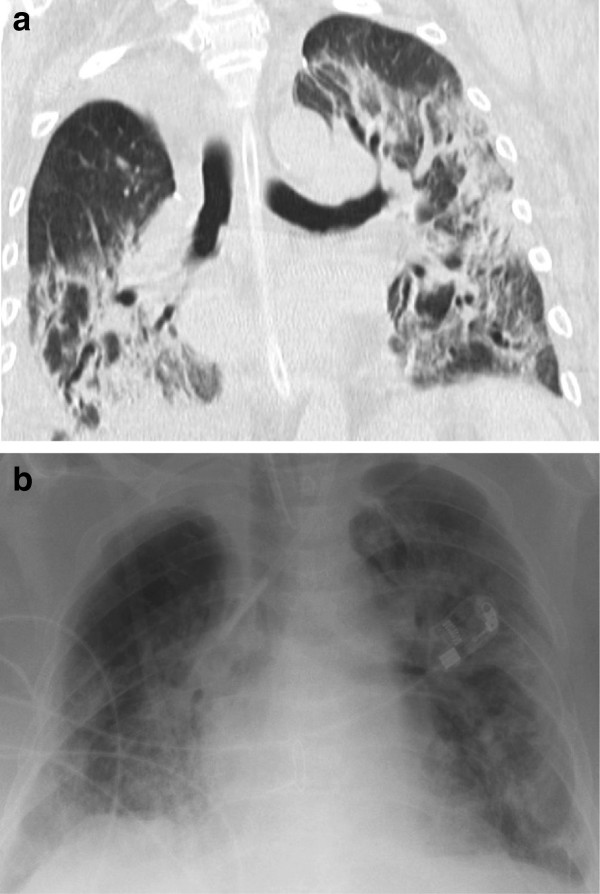
Radiographic findings: computer tomography shows bronchiectasis and consolidation especially in the right lower lobe (a) and increasing bilateral diffuse pulmonary infiltrates in both lungs with ground glass opacities seen in chest x-ray (b).

Chest radiography revealed increasing bilateral diffuse pulmonary infiltrates in both lungs with ground glass opacities. Additionally, bronchiectasis and consolidation in the right lower lobe were noticed (Figure 1).

### Infectious workup

In sputum and BAL samples, infection with influenza virus A/H1N1 could be verified four days prior to death by real-time RT-PCR [1]. Multiplex-PCR proved to be negative for other bacterial or viral infections. Accordingly, cytomegalovirus PCR, cell culture assays and bacterial and fungal cultures were negative during her last hospitalisation period at the University Medical Center Freiburg.

### Post mortem

**Figure 2:**
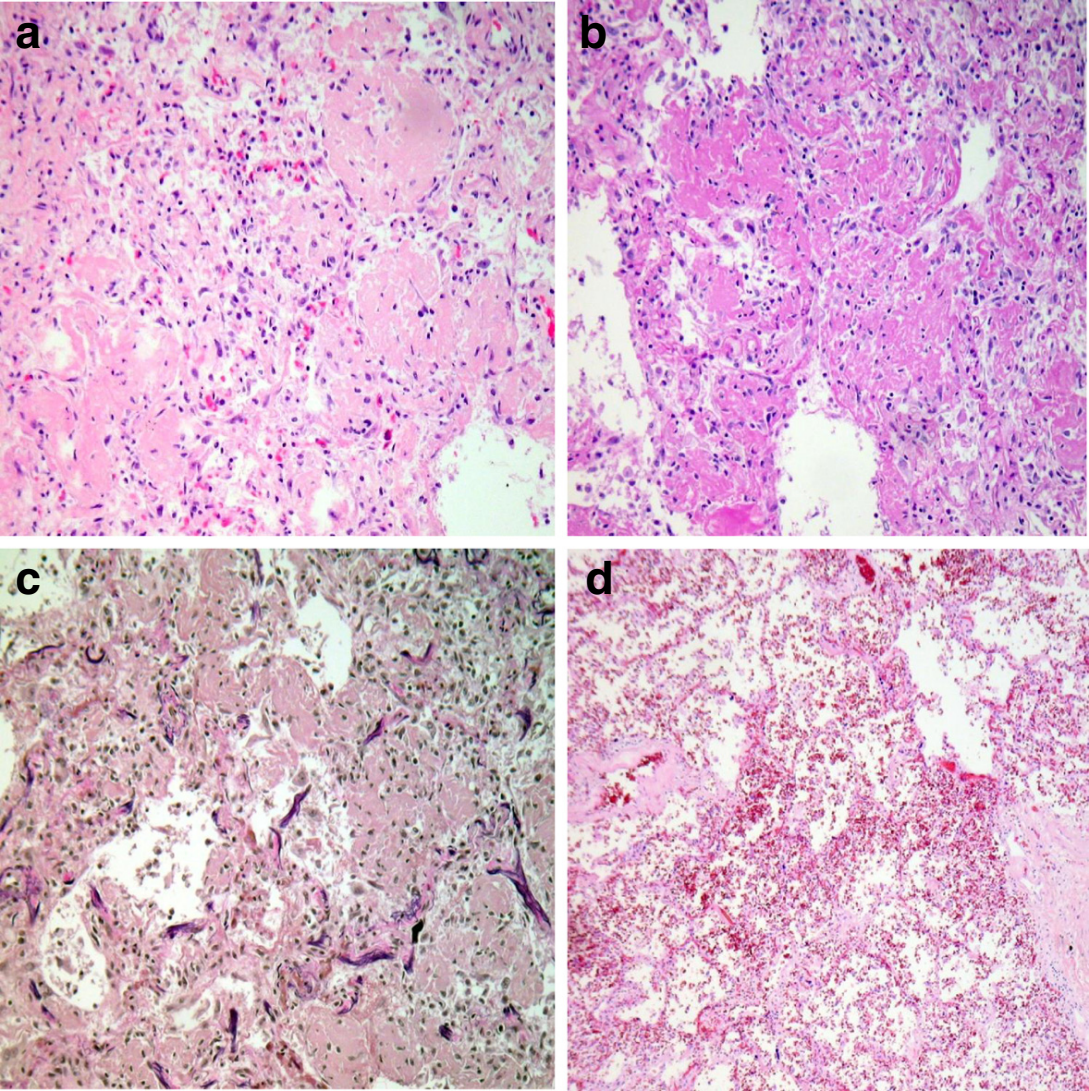
**Postmortem histological findings in the lungs. a**-**c**) AFOP: patchy involvment of lung parenchyma of the lower lobe with cubic intraalveolar fibrin deposits (so called fibrin balls) and formation of fresh fibroblast foci as sing for organizing pneumonia, hyaline membranes are absent (10×; H&E, PAS and EvG staining); **d**) unaffected lung parenchyma of the upper lobe (10×; H&E staining).

Thoracic situs with status post double sided lung transplantation was evident with sufficient anastomoses of the transplanted organs. The lungs were consolidated in consistency but patchy discolorization typical for bacterial pneumonic infections were not present. Especially the lower parts of the lungs showed classical microscopic features of AFOP with distinct patchy intraalveolar formation of fibrin balls, focal fibroblast foci and diffuse organizing pneumonia (Figure 2). Hyaline membranes were not detected in any histological specimens. Thus, pure AFOP not associated with other histological patterns of DAD was verified. No signs of graft rejection, bacterial or fungal superinfections or a relapse of UIP were seen.

Molecular analysis of lung tissue revealed positive results for influenza A/H1N1by PCR. There was no detection of any bacterial or fungal infection in any analysed lung specimen, neither by microbiological nor by histological examination.

## Discussion

Epidemic influenza A/H1N1 originally developed in the United States of America and Mexico in 2009 and finally spread worldwide to a pandemic disease [2,3]. The first case in Germany was registered in April 2009 [4]. Influenza virus infections may cause serious pulmonary complications with death rates of approximately 7% [5,6]. Although previously healthy patients may suffer from severe H1N1 infection, mortality proved to be particularly high in patients with predisposing conditions, i.e. obesity, pulmonary diseases, other than asthma or chronic obstructive pulmonary disease, pregnancy or pneumonia [6].

Patients with primary or secondary immunodeficiencies, particularly patients after lung transplantation are at especially high risk for development of severe pulmonary complications caused by influenza A/H1N1 infections. This, therefore, demonstrates a severe condition in lung transplanted patients [2,5,7].

AFOP was first described by Beasley et al. [8] in 2002 and represents a distinct histological pattern of lung injury related to DAD. Beasley described two forms of AFOP: one less aggressive with good response to corticosteroids, the other with poor clinical outcome and a high mortality rate of 53% [8]. AFOP presents with specific histological features, i.e. patchy intraalveolar fibrin deposits, diffuse organizing pneumonia and absence of hyaline membranes. In the current literature, causes of AFOP are idiopathic or in combination with collagen vascular diseases [9], bacterial infections [10] and after certain drug exposure (e.g. amiodarone, abacavir [8,11], statins [12] as well as exposure to inhalative agents (e.g. coal mining, construction, zoological work, hairspray) [8].

One of a newborn child is reported with acute respiratory distress syndrome and AFOP after respiratory syncytial virus pneumonitis [13]. However, histology revealed hyaline membranes in the vicinity of AFOP-patterns, thus classifying it as DAD with focal patterns of AFOP. In contrast, upon autopsy of our patient no hyaline membranes were detected in any tissue sample. Therefore, in the case presented here, pure AFOP in association with viral infection is to be diagnosed.

## Conclusion

Concluding, for the first time proof is given that viral agents at least of the myxovirus family - here influenza A/H1N1 - can induce pure often fatal AFOP. Consequently, AFOP in the setting of viral pneumonia must be taken into account especially in immunocompromised patients as successful treatment with a combination of corticosteroids and mycophenolate-mofetil has recently been reported [14].

## Consent

Informed and written consent for post-mortem investigation, scientific research and following publication as clinical image was given by the patient prior to death.

## Competing interests

The authors declare that no competing interests exist.

## Authors’ contributions

CO, GK: autopsy workup, preparation of the manuscript. DH: virological investigations, preparation of the manuscript. LK: radiological evaluation, preparation of the manuscript. CB, AH, SR, TP: clinical care, provision of clinical data, proof reading. MW: Histological evaluation, methodological support, proof reading. All authors read and approved the final manuscript.

## Pre-publication history

The pre-publication history for this paper can be accessed here:

http://www.biomedcentral.com/1471-2466/13/30/prepub
